# Social Research on Neglected Diseases of Poverty: Continuing and Emerging Themes

**DOI:** 10.1371/journal.pntd.0000332

**Published:** 2009-02-24

**Authors:** Lenore Manderson, Jens Aagaard-Hansen, Pascale Allotey, Margaret Gyapong, Johannes Sommerfeld

**Affiliations:** 1 Faculty of Medicine, Nursing and Health Sciences, Monash University, Clayton, Victoria, Australia; 2 DBL - Centre for Health Research and Development, University of Copenhagen, Copenhagen, Denmark; 3 Centre for Public Health Research & School of Health Sciences and Social Care, Brunel University, West London, Uxbridge, United Kingdom; 4 Dodowa Health Research Centre, Ghana Health Services, Dodowa, Greater Accra Region, Ghana; 5 UNICEF/UNDP/World Bank/WHO Special Programme for Research and Training in Tropical Diseases (TDR), World Health Organization, Geneva, Switzerland; Ghana Health Services, Ghana

## Introduction

Neglected tropical diseases (NTDs) exist and persist for social and economic reasons that enable the vectors and pathogens to take advantage of changes in the behavioral and physical environment. Persistent poverty at household, community, and national levels, and inequalities within and between sectors, contribute to the perpetuation and re-emergence of NTDs. Changes in production and habitat affect the physical environment, so that agricultural development, mining and forestry, rapid industrialization, and urbanization all result in changes in human uses of the environment, exposure to vectors, and vulnerability to infection. Concurrently, political instability and lack of resources limit the capacity of governments to manage environments, control disease transmission, and ensure an effective health system. Social, cultural, economic, and political factors interact and influence government capacity and individual willingness to reduce the risks of infection and transmission, and to recognize and treat disease. Understanding the dynamic interaction of diverse factors in varying contexts is a complex task, yet critical for successful health promotion, disease prevention, and disease control. Many of the research techniques and tools needed for this purpose are available in the applied social sciences. In this article we use this term broadly, and so include behavioral, population and economic social sciences, social and cultural epidemiology, and the multiple disciplines of public health, health services, and health policy and planning. These latter fields, informed by foundational social science theory and methods, include health promotion, health communication, and heath education.

Social science health researchers have attended particularly to HIV/AIDS, and more recently to malaria and tuberculosis (TB), reflecting the prevalence and resistance to control of these diseases and their emphasis in the United Nations Millenium Development Goals. Other infectious diseases, by default, have slipped into a “neglected” category. These include most “tropical” diseases, such as Chagas disease, dengue, human African trypanosomiasis, leishmaniasis, leprosy, lymphatic filariasis, schistosomiasis, and onchocerciasis. The inclusion of these diverse diseases as “neglected” refers not only to their status relative to HIV, TB, and malaria. Their neglect reflects their epidemiology: they are prevalent among the poorest and most marginalized of the world's population. More than 70% of countries and territories affected by NTDs are low-income and lower middle-income countries, and 100% of low-income countries are affected by at least five NTDs [1]. This is due to multiple factors, including the focality of most NTDs and hence the localization of vulnerability, morbidity, and mortality. Various social determinants (e.g., poverty, gender, education, and migration) interact to establish local patterns of co-morbidity of NTDs and other pertinent public health problems (e.g., malnutrition, malaria, diarrheal diseases, and violence). These vulnerable populations tend to lack the power to draw attention from decision makers to their problems and to attract resources, and national resources tend to be directed to high prevalence, epidemic conditions at the expense of endemic diseases. NTDs also attract little research nationally or internationally, and virtually no investment or commercially based research and development in wealthy research settings [2].

In recent years, however, NTDs have received increasing international interest, partly in response to promising advances in drug development. Concerted efforts are being made also to promote innovative public health approaches such as integrated delivery of multiple interventions [3]–[5], which require research effort into effective public health interventions. This article was stimulated by the renewed interest in populations affected by NTDs and in feasible ways to prevent and control NTDs. Rather than focusing on specific medically defined NTDs, in this article, we focus on neglected diseases of poverty, i.e., diseases that disproportionately affect poor and marginalized or, in other words, diseases of “neglected” populations. We begin with a summary of the history of social research activities supported by the Special Programme for Research and Training in Tropical Diseases (TDR) at the World Health Organization (WHO). We then highlight the ongoing and emerging challenges to sustain and extend research to improve the control of NTDs, all of which are also neglected diseases of poverty. We identify emerging research priorities and reflect on the challenges in mainstreaming these issues in research and disease control programs, drawing attention to the urgency of particular research questions.

## Methods

The focus of this review was established at an expert consultation in which we participated, hosted by TDR on April 23–24, 2007. The experts convened to examine the current status of applied social science research in tropical disease control, identify ongoing challenges, and develop a strategy to mainstream gender and the social sciences within TDR. Priority areas for the review were based on consensus panel discussion. Literature reviewed was identified through MeSH heading searches in Web of Science, PubMed, and Scopus using various combinations of terms including social science, tropical diseases, neglected diseases, gender, and poverty. The review also drew on research funded by TDR and work conducted by TDR-trained scientists, represented both in peer-reviewed journals and in grey literature. Our aim was to inform developments in the identified key areas, and reflecting this aim, we have not sought to cover comprehensively all social science research in tropical and neglected diseases. In addition, we do not necessarily reflect the views of WHO, nor specifically those of TDR.

### The Evolution of Themes in Social Research on NTDs

The research themes and priorities for social research on NTDs reflect evolving approaches and discourses in international public health and the specific public health challenges of the era. In the early 1950s, well before the international health community would coin the term “neglected tropical diseases,” public health practitioners involved in infectious disease control programs had developed a keen interest in applied social science research. Considerable work began to focus on understanding reasons for adverse reactions to vertical infectious disease control efforts [6]. Later, notable advances in medical anthropology led to health social science applications in health education and community participation [7]–[9].

In the 1970s, primary health care, community participation, and support for horizontal health care systems emerged as important concepts and tools to address health inequalities. TDR was established as a joint special program of the United Nations Development Programme (UNDP), the World Bank, and WHO (now with the partnership also of the United Nations Children's Fund [UNICEF]) to counteract the neglect in research and development efforts for tools to combat infectious diseases among the poor [10],[11]. The program recognized not only the impact of infectious diseases in undermining people's health but also the links between economic development, poverty alleviation, and good health. In 1976, anticipating the emphasis on community and society iterated at the Alma Ata Conference (1978), the then Director-General of the WHO, Dr. Halfdan Mahler, emphasized that the “(TDR) Programme was not designed simply to advance medical technology but rather as a contribution to the promotion of human welfare in the widest sense, in the context of a new international order in economic and social affairs.” The first technical review group called for “a commitment to long-term continuity of (such) research, which had been lacking from most previous efforts in the field.” Preparations to do this took two years because of the absence of a social research community and significant relevant research tradition on which to build; the first TDR Steering Committee on Social and Economic Research became operational in late 1979.

TDR funded a significant number of young scholars for higher degrees as well as an expanding number of research projects through the Research Strengthening Group and the Steering Committee on Social and Economic Research, and after 1994, through various initiatives and task forces on applied field research [12]. These committees oversaw the development of methods and basic research to describe the effects of poverty, gender, quality of care, and other socio-cultural contexts on exposure, experience, health-seeking behaviors, and sequelae of disease. They included projects concerned with interventions, with particular attention to the potential merit of social science information to national control programs and nongovernmental organization and private sector interventions. This work included the development of rapid assessment tools for malaria [13]–[16], the use of school-based surveys to assess community prevalence of schistosomiasis [17]–[22], the establishment of economic analyses of tropical disease research and interventions [19], [23]–[26], the development of gender-sensitive health services interventions [27]–[31], and the implementation of collaborative work on the household management of fever to support the early diagnosis and treatment of malaria and pneumonia [32]–[34]. Important social research breakthroughs resulting from field research initiatives included the development of the concept of community-directed treatment for onchocerciasis, insecticide-treated bednets, and development of unit dose packaging (blister packs) for easy distribution of anti-malarials to communities and homes — interventions that empowered community members to take simple measures on their own to prevent disease and protect their health. The research programs developed from 1979 to the mid 1990s highlighted a commitment by collaborating researchers in the concerted and systematic application of trans-disciplinary social sciences in tropical disease research and control programs [35]–[38].

Over the decades, a considerable sub-literature on social sciences in infectious diseases and their control has emerged, including chapters in textbooks of tropical medicine [39]–[41], resulting in significant bodies of evidence in health economics, health policy research, and (medical) anthropology of infectious diseases [42]. A missing and critically needed perspective in research was the foregrounding of gender [27], [38], [43]–[53]. The link between gender and exposure, risk, susceptibility, disease experience, and outcome was established through a number of studies, based on secondary analyses of quantitative data and new qualitative studies explicitly concerned with gender and its impact on vulnerability and outcome. These studies highlighted differences in rates of infection tightly correlated with economic activities and social status, and drew attention to significant disparities in access to treatment. This work resulted in the increased collection and reporting of sex disaggregated data, and increased attention to the effects of both sex and gender on disease. Research on female genital schistosomiasis, relationships for women between stigma and treatment, and gender inequalities in access to resources and presentation for care provide powerful examples of developments in this area [47], [52], [54]–[62].

In the mid 1990s, “upstream” issues such as globalization, equity, gender, and human rights gained increasing prominence in international health. In 2000, a new TDR Steering Committee on Social, Economic, and Behavioural Research (SEB) was established with the mandate to build on, promote, and support social research identifying constraints in, and opportunities for, infectious disease control and prevention in resource-poor settings. Emphasis was placed on elucidating social, cultural, economic, health-systems, and policy-related factors, and proposing strategic solutions to barriers in disease control and public health. In contrast to the work of the earlier committees, attention now was placed on social research that would address large-scale, “transnational” issues and challenges in relation to infectious diseases and their control. Researchers were encouraged, in this context, to attend to the societal and economic impact of globalization as well as specific disease and health-systems factors [12]. A clearer elucidation of globalization led to research on the impact of widening social inequalities on disease persistence, emergence, and resurgence [63]; the effects of political conflict and other forms of violence on NTDs; the role of community resilience; the ethical, legal, and social implications of biotechnology use and transfer into resource-poor settings [64]; and a human rights analysis of NTDs [65]. Research with a sharper focus on public health systems in endemic countries focused on equity effects of health sector reforms [66], research ethics [67], and inequalities of access to proven therapies, prevention, and information. Research in health economics focused on human resources, including difficulties in sustaining the health research workforce and retaining both volunteers and health system staff [68]. While some research was also conducted on private sector collaboration and emerging interest in public–private partnerships (PPPs) [69], this has been generally limited because of poorer investment in research [70]. TDR's social research activities address both basic social science and implementation research issues, including most recently research on community-directed interventions for major health problems in Africa [71].

The research programs and related training of social scientists have consolidated the role of social sciences in the tropical disease agenda, particularly with respect to a stronger evidence base on the social determinants of health, on potential areas for interventions, and on preliminary developments in the area of implementation research. However, major challenges remain in understanding the complex interactions of community, household, personal, and governmental factors that maintain health and produce disease, and in finding effective ways to address these issues at various political levels.

### Continuing and Emerging Themes

As reflected in the bibliography, the social science and applied health literature on infectious diseases of poverty is substantial, but uneven across diseases, themes, regions, and institutions. There is, for example, greater attention to communities who are vulnerable to disease, and less to institutions involved in disease prevention and control. Below, we draw attention to what we regard now as the most urgent and emerging research questions.

### Government, Community, and Environmental Change

Continued research is needed on the implementation of interventions and control programs to ensure a critical evidence base to inform the effective, sustained, and embedded adoption of interventions by communities [72]. This involves a more critical understanding of government decision-making and individual choices related to disease prevention, and a better understanding of how the relationships of people to their governments influence adherence, shared commitment and community participation in control programs [73],[74]. Since the Alma Ata Declaration of 1978, there has been considerable interest in community involvement, volunteer activities, relationships between local governments and communities, and decentralization. Early work focused on the ways in which these approaches might work to control infectious disease in rural areas, where there were almost always limited resources, poor infrastructure, and lack of services. But the prevalence of infectious diseases in urban areas has become an increasing concern, reflecting global trends in urbanization and the inability of urban as well as rural governments to manage infrastructure and meet the health and welfare needs of their populations. Conflict usually results in or contributes to a breakdown of health services infrastructure and migration of vulnerable populations, often with a negative impact on the control of NTDs.

Increased urbanization is partly driven by economic changes, but also by environmental and climate change, resulting in changed patterns of land use and residence, and changes in vector habitat and behavior. Global warming has both direct and indirect effects on the distribution and prevalence of NTDs, highlighting the need for further research on the links between society, environment, agriculture, and human health, and the relationship of these factors to the control of neglected vector-borne diseases such as dengue [46],[75]. Water resource development schemes often lead to new exposure of vulnerable populations, and health impact assessments based on social science approaches are critical. Further research is also needed on community participation in the prevention and control of disease in urban and peri-urban slums; on the social organization of urban areas to establish mechanisms for the implementation of community-directed treatment approaches in cities; and on vertical versus horizontal approaches and effective implementation of interventions under decentralization.

Notwithstanding growing attention to health programs, health services, and access to care, research is still required to explore how access to health services is conditioned by poverty and inequality, as shaped by structural and political-economic factors (gender, ethnicity, migration patterns, etc.). In an emerging research agenda, there is a need to move to explore practical ways to disrupt disease transmission and enhance accessibility of care. Because of changes in land use, climate, and population demographics, and subsequent changes in the distribution of NTDs and continuing risk of drug resistance, there is a need too for ongoing research on the maintenance of disease control in areas of low prevalence. This will enable monitoring and prevent resurgence, without the need for resource-intensive programs. The various roles of the not-for-profit sector, industries, and civil society need further exploration. There are also continuing questions regarding government and population interactions, governance and government institutions [73],[76].

The research on gender has almost without exception focused on issues affecting women with a disregard for how gender affects the disease experience of men. The fluid nature of the concept of gender and its dynamic interaction with other determinants of vulnerability, such as socioeconomic status, ethnicity, and age, also remain poorly understood. Our understanding of the significance of and interactions between gender differences and other social and economic variables is sparse, and little work has been conducted to apply our current knowledge from gender studies to the development of gendered policy and practice across all aspects of the health sector, including human resources and capacity building. These issues need to be understood within a broader political and environmental context that takes into account issues such as inequality, political instability and violence, displacement, and globalization [77].

### Biomedicine and Innovation

New biomedical priority areas need to be enhanced by social science research. Innovative vector control interventions and new drugs and diagnostics need to be considered in terms of their introduction, acceptability, and adherence, and the integration of such innovations as a component of community-based interventions. Research needs to be undertaken on the acceptability and utilization of drugs in multi-intervention approaches for disease control (e.g., combined use of praziquantel and oxamniquine), including in relation to the acceptability and affordability of new approaches and new drug regimes. People in endemic areas frequently have multiple infections; however, limited work has been undertaken on the social implications of this. Other areas requiring greater attention include decision-making regarding treatment, the impact of complex treatments, particularly when a person has more than one communicable and/or non-communicable disease, willingness to carry the cost of treatment for recurrent infections, and attitudes towards side effects.

Research needs to be continued on the supply and distribution of drugs, including in relation to the proliferation of counterfeit drugs, the failure or inability to adhere to prescribed treatment regimes, the illegal circulation of drugs, and other questions on the use of pharmaceuticals and the roles of the private sector [78]. With the increase in large-scale drug-based, multi-disease control programs, it is necessary not only to monitor pharmacological side effects (“pharmaco-vigilance”), but also to understand evolving attitudes in the target populations (“socio-vigilance”). A number of NTDs, particularly helminthic infections, leprosy, and in India, Nepal, and Bangladesh, visceral leishmaniasis, have the potential to be eliminated. To support this effort, further work is needed on cost-effective strategies using optimal interventions that include both treatment of disease and where applicable, vector control.

TDR's social research activities address both basic social science and implementation research issues, including most recently research on community-directed interventions for major health problems in Africa [71]. Political and economic changes, with or without violence as a backdrop, influence the willingness of populations to trust in and collaborate with disease control agencies, and their preparedness to develop common goals for disease prevention. Again, the relationship between communities, householders, and the public and private sectors, and the optimal ways of bringing these together, needs to be explored. Strategies are required to extend integrated disease control programs for NTDs and malaria in areas where community-directed treatment programs are established, as in onchocerciasis control areas.

### Capacity Building and Managerial Issues

The hierarchical structure of personnel within the health sector in many disease-endemic countries stems from a colonial legacy that privileges the knowledge and contributions of biomedically trained personnel, and fails to appreciate fully the importance of engaging with a range of health professionals, such as lay providers, volunteer workers, and traditional specialists, to enhance the effectiveness of behavioral, household, and community-based interventions [72]. There is a need to pursue the integration of NTD control and routine primary health services [79] and to analyze the reasons why NTD prevention activities and outreach receive low priority [80],[81]. It is clear, for instance, that health sector reform has not produced a uniform community gain, and those who are most vulnerable to NTDs are often hardest hit [82]. PPPs have been proposed as an alternative. However, the relationships between public and private providers, and the viability of this approach in different settings, is complex, partly because of different interests and commitments to disease control [83]. Reflecting this, there has been limited investment in social science and health systems research on private sector collaboration with disease control programs [70].

While social scientists need to engage in research related to health policy, administration, and management, in countries where NTDs are endemic, there are still few applied social scientists working on health-related questions, and a limited understanding within the health sector of the contributions that they might make. Further, for non-medically trained health service personnel engaged in research and in the design and delivery of programs, there is usually a limited career trajectory: social scientists are typically employed at levels not commensurate with their qualifications, without opportunities to utilize their specialized skills. This lack of recognition serves as a disincentive for those with the capacity to return to or remain within the health sector, contributes to their dissatisfaction in improving health services, limits the quality of applied social science research, and inhibits the translation of relevant social science findings into practice.

## Conclusion

The research themes that we believe to be of key importance in the years to come fall into two broad areas. One relates to globalization and its impacts: global warming and changes in the epidemiology of disease, urbanization, anthropogenic environmental change, and the availability, cost, and distribution of drugs. The other area relates to the control of disease, and in this context, to community participation, government–community partnerships, PPPs, health services research, and strategies for control of both single diseases and multiple infectious diseases. It will take time to nurture and strengthen new areas of research, not least if they are breaking new ground and are conceptually difficult; it will also take time, and is always complex, to sustain the small group of researchers working in these fields in endemic countries. Strategies and resource allocation need to be based on long-term outcomes.

NTDs are referred to often as diseases of poverty, but implicit in the use of the term poverty is the tight inter-relationship of poverty and inequality. This reference to poverty extends to include individuals, households, communities, and countries. It refers to the individuals and households affected by infectious diseases, the effects of continuing, untreated infection, and the impoverishment that occurs as a direct result of disease and the high costs of health care. It refers to the material circumstances of communities at risk—in poor, isolated, and ill-served rural areas and in the sub-standard conditions of urban slums and squatter settlements. It acknowledges, too, the difficulties faced by countries too poor to provide the infrastructure, human resources, and services that reduce the toll of such infections, and that are crippled by international debt and economic disadvantage in ways that are echoed in the incidence and prevalence of diseases. A social science perspective on diseases of poverty is critical to ensure that equity remains an underlying principle in policy development, research, advocacy/dialogue, legislation, resource allocation, planning, implementation, and monitoring of programs and projects.

**Box 1. Key Learning Points**

- Social research has drawn attention to the difficulties in ensuring effective and sustained interventions for NTDs in both urban and rural communities, and in environments that have been disrupted by war, resettlement, and migration.
- Gender has a major impact on the distribution of disease, risks of transmission, and diagnosis and patterns of care. However, the links between gender differences and other social and economic variables, such as socioeconomic status, ethnicity, and age, are poorly understood.
- Social research on community diagnosis, treatment, and control highlights the importance of community participation for the successful introduction, acceptability, and adherence of innovative vector control interventions and new drugs and diagnostics.
